# EXPath: a database of comparative expression analysis inferring metabolic pathways for plants

**DOI:** 10.1186/1471-2164-16-S2-S6

**Published:** 2015-01-21

**Authors:** Chia-Hung Chien, Chi-Nga Chow, Nai-Yun Wu, Yi-Fan Chiang-Hsieh, Ping-Fu Hou, Wen-Chi Chang

**Affiliations:** 1 College of Biosciences and Biotechnology, Institute of Tropical Plant Sciences, National Cheng Kung University, Tainan 70101, Taiwan

## Abstract

**Background:**

In general, the expression of gene alters conditionally to catalyze a specific metabolic pathway. Microarray-based datasets have been massively produced to monitor gene expression levels in parallel with numerous experimental treatments. Although several studies facilitated the linkage of gene expression data and metabolic pathways, none of them are amassed for plants. Moreover, advanced analysis such as pathways enrichment or how genes express under different conditions is not rendered.

**Description:**

Therefore, EXPath was developed to not only comprehensively congregate the public microarray expression data from over 1000 samples in biotic stress, abiotic stress, and hormone secretion but also allow the usage of this abundant resource for coexpression analysis and differentially expression genes (DEGs) identification, finally inferring the enriched KEGG pathways and gene ontology (GO) terms of three model plants: *Arabidopsis thaliana, Oryza sativa, and Zea mays*. Users can access the gene expression patterns of interest under various conditions via five main functions (Gene Search, Pathway Search, DEGs Search, Pathways/GO Enrichment, and Coexpression analysis) in EXPath, which are presented by a user-friendly interface and valuable for further research.

**Conclusions:**

In conclusion, EXPath, freely available at http://expath.itps.ncku.edu.tw, is a database resource that collects and utilizes gene expression profiles derived from microarray platforms under various conditions to infer metabolic pathways for plants.

## Background

Plants, which are classified as the kingdom Plantae, provide source of energy and oxygen in ecosystems and the majority of agricultural production worldwide. To maintain the autotrophic mechanisms as well as the resistance to impacts from surroundings (e.g., extreme weather, soil salinity, and pests), the elaborate control of gene expression and collaboration under various environments or conditions at molecular level is critical and related to growth, development, and the yield of crop production in plants [1]. Since the lack of motility compels plants to be more tolerant against the threat of external stresses, genes involved in stress-related response, signal transduction pathways, and the induced transcription factors (TFs) were progressively discovered through the comparative genomics approaches [2-5]. Moreover, phytohormones, which are believed to modulate plant growth and diverse development processes, have been reported in relation to environmental variation in *Arabidopsis *and maize [6-8]. The evidence reveals that the complexity of gene regulation in significant pathways or biochemical reactions plays an important role in coping with plants survival and their self-defense mechanisms towards different circumstances.

In general, the expression of gene alters conditionally to catalyze a specific metabolic pathway [9,10]. Comprehensively investigating how genes are activated or repressed, i.e., differentially expressed genes (DEGs), in vital biological processes under various conditions are essential to understand gene functions and the coexpression manner in metabolic routes. In recent decades, microarray-based datasets have been massively produced to monitor gene expression levels in parallel with numerous experimental treatments [11]. This high-throughput detection of transcript quantity facilitates the comparative expression analysis by combining multiple microarray expression data among different samples and even different species [12]. Due to the abundance of expression datasets generated by microarray platforms for plants, a plenty of databases and resources have promptly collected gene expression data that are publicly accessible. Among them, Gene Expression Omnibus (GEO) provides most profuse microarray expression datasets presented with the function of GEO DataSets, GEO Profiles, and GEO2R Analysis [13]. Although GEO2R Analysis allows users to compare multiple expression data and then identify DEGs, GEO Profiles can only display the expression level of one gene across different samples in each dataset. Another powerful tool, eFP Browser, is easily adaptable for analyzing microarray or other large-scale datasets in plants by using pictographic representations [14]. Additionally, PLEXdb, GENEVESTIGATOR, NASCArrays, and RiceXPro are also useful repositories for microarray gene expression profiles in *Arabidopsis*, rice, and plants [15-18]. On the other hand, to gain a comprehensive insight into plant metabolic pathways that are consisted of metabolites and enzymes, relevant databases were established recently. Gramene, a comparative resource for plants, summarizes ten databases of plant metabolic pathways, e.g., AraCyc, RiceCyc, MaizeCyc, BrachyCyc and SorghumCyc [19]. Moreover, MetNet Online integrates information of metabolic pathways and regulatory networks for *Arabidopsis thaliana*, *Glycine max *and *Vitis vinifera *[20]. Other instances of similar pathway knowledge bases are Arabidopsis reactome and Pathway studio [21,22].

**Figure 1:**
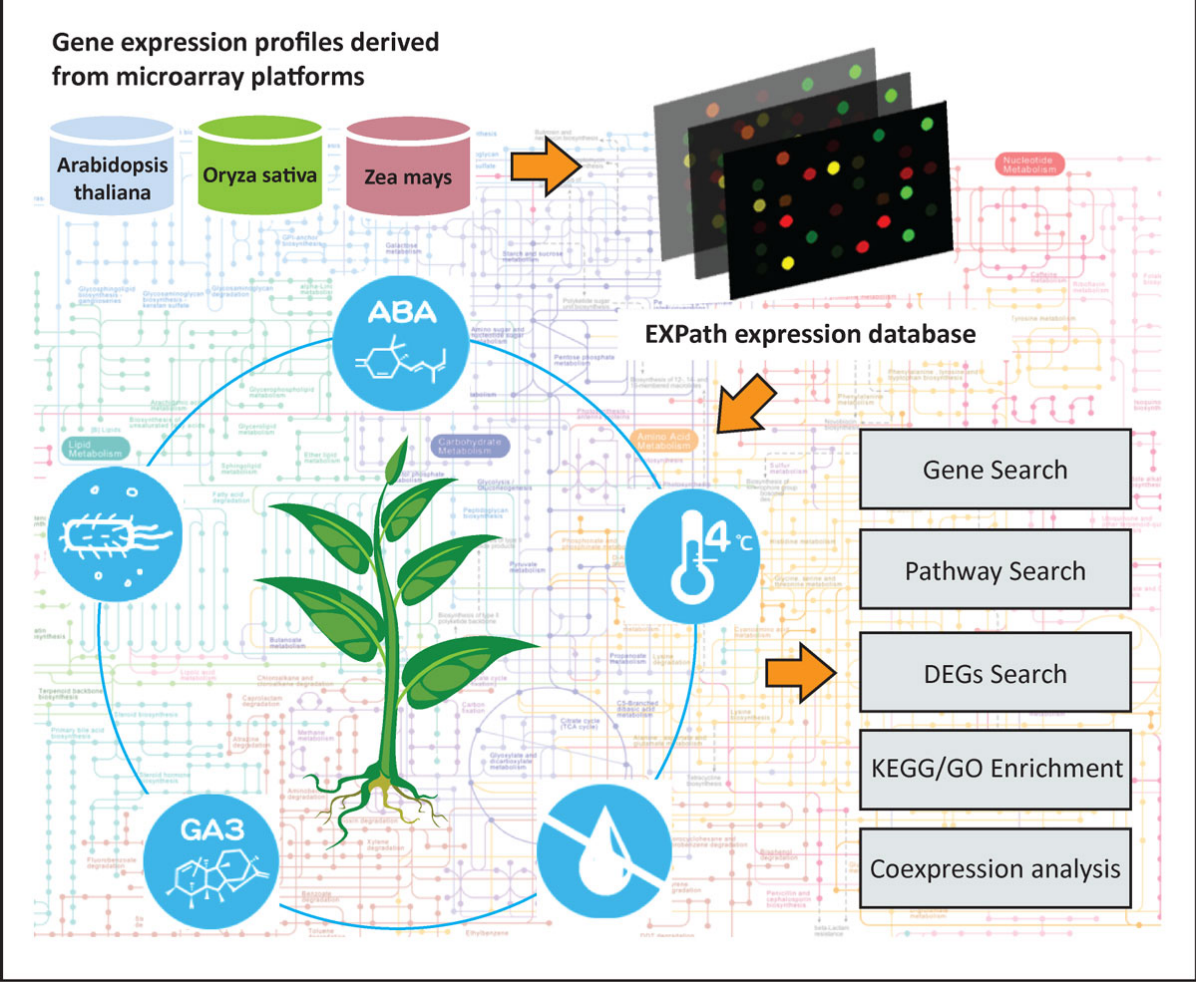
**The concept and construction of EXPath database**. Five main functions (Gene Search, Pathway Search, DEGs Search, Pathways/GO Enrichment, and Coexpression analysis) and the advanced combination analysis of them are provided.

To estimate the expression level of each gene involved in significant biological processes thoroughly, it is important to integrate gene expression data with metabolic pathways. To our current knowledge, Jensen and Papin have presented a method, Metabolic Adjustment by Differential Expression (MADE), for mapping expression data onto a metabolic network model without using arbitrary expression thresholds. Unfortunately, MADE is implemented in Matlab and only supports for *Saccharomyces cerevisiae *[23]. Another web-based tool, Array2KEGG, attempted to depict up or down regulated genes in a particular KEGG pathway image of interest. However, the system were developed for human, mouse, and rat. Furthermore, although Pathway Processor 2.0 is a web resource for converting gene expression into pathway expression and identifying differentially regulated pathways in an input datasets, users have to submit their own expression data, and cannot be apply for plants [24]. Besides, advanced analysis such as pathways enrichment or how genes express under different conditions is not rendered. It is noted that AlgaePath that we published previously makes feasible to analyze metabolic pathways using transcript abundance data from next-generation sequencing in green algae [25], but it is created for non-vascular plants. Therefore, EXPath was developed to not only comprehensively congregate the public microarray expression data from over 1000 samples in biotic stress, abiotic stress, and hormone secretion but also allow the usage of this abundant resource for coexpression analysis and DEGs identification, finally inferring the enriched KEGG pathways and gene ontology (GO) terms of three model plants: *Arabidopsis thaliana*, *Oryza sativa*, and *Zea mays*. Users can access the gene expression patterns of interest under various conditions via five main functions (Gene Search, Pathway Search, DEGs Search, Pathways/GO Enrichment, and Coexpression analysis) in EXPath, which are presented by a user-friendly interface and valuable for further research. The concept and construction of EXPath is illustrated in Figure 1.

## Construction and content

### Repository for microarray gene expression data

To establish overarching repository for gene expression in plants, EXPath curates 1057 samples treated with biotic stress, abiotic stress, and hormone secretion from publicly available microarray gene expression data for *Arabidopsis thaliana*, *Oryza sativa*, and *Zea mays*. Among them, expression profiles of *Arabidopsis thaliana *were retrieved from AtGenExpress [26] and NASCArrays [18], whereas others were obtained via RiceXPro (*Oryza sativa*) and GEO (*Oryza sativa *and *Zea mays*) [13,16]. Table 1 summarizes the categories and number of microarray samples collected in EXPath expression database.

**Table 1:** Categories of microarray samples in EXPath expression database.

Species	Stresses		# of samples	Resource
***Arabidopsis thaliana***	Abiotic stress	Cold stress	298	AtGenExpress [26] NASCArrays [18]
		Drought stress		
		Genotoxic stress		
		Heat stress		
		Osmotic stress		
		Oxidative stress		
		Salt stress		
		UV-B stress		
		Wounding stress		
	Biotic stress	*Phytophthora infestans*	108	
		*P.syringae pv.tomato *DC3000		
	Hormones	ABA	96	
		Auxin		
		Brassinolide		
		Brassinosteroids		
		GA		
		Gibberellin		
		Jasmonic acid		
		Zeatin (cytokinin)		
***Oryza sativa***	Abiotic stress	Cold stress	94	RiceXPro [16] GEO [13]
		Salt stress		
		Drought stress		
	Biotic stress	*Agrobacterium tumefaciens*	161	
		*Magnaporthe oryzae*		
		*Magnaporthe oryzae strain Guy11*		
		*Meloidogyne graminicola*		
		*Striga hermonthica*		
		*X. oryzae pv. oryzae*		
		*X. oryzae pv. oryzicola*		
	Hormones	Abscisic acid	138	
		Auxin		
		Brassinosteroid		
		Cytokinin		
		Gibberellin		
		Jasmonic acid		
***Zea mays***	Abiotic stress	Acid soil	64	GEO [13]
		Drought		
		Waterlogging		
	Biotic stress	*Colletotrichum graminicola*	92	
		*Meloidogyne incognita*		
		*Phytophthora cinnamomi*		
		*Sporisorium reilianum f. sp. Zeae (Kühn)*		
		*Ustilago maydis*		
	Hormones	GA3	6	
		IAA		

### Data processing and normalization

**Figure 2:**
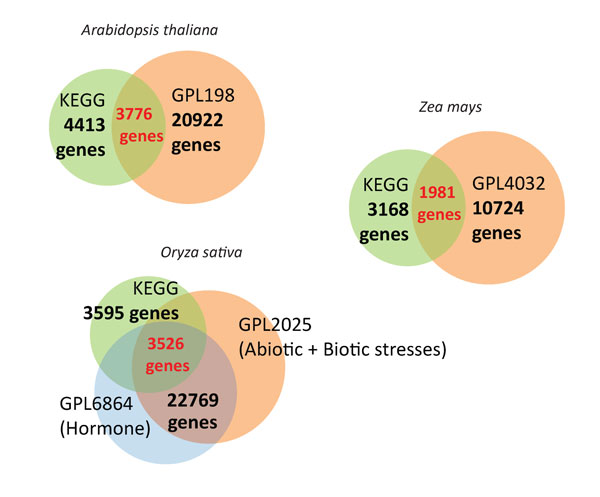
**The statistics of genes with valid expression data in EXPath**. In total, 20922 genes for *Arabidopsis thaliana*, 22769 genes for *Oryza sativa*, and 10724 genes for *Zea mays *were congregate respectively. The number of genes overlapped with KEGG annotated genes are shown in red.

Since the platforms for inclusive microarray datasets in EXPath are either Affymetrix GeneChips (GPL189, GPL2025, and GPL432) or Agilent Technologies (GPL6864), probe set annotation data in tabular format were used to map probe set ids to detectable genes. For the purpose of optimizing input data for comparative expression analysis, ambiguous measurements of gene expression, for example, probes associated with more than two genomic loci or control probes, were discarded. In total, 20922 genes for *Arabidopsis thaliana*, 22769 genes for *Oryza sativa*, and 10724 genes for *Zea mays *were congregate respectively. Moreover, genes matched with KEGG genes and KEGG orthology (KO) were also calculated. The statistics of genes with valid expression data in EXPath is displayed in Figure 2.

After processing the raw data of microarray datasets, the normalization procedure was executed to avoid systematic biases arising from the variation between different trials (GEO series/GSEs) and samples. In this work, robust multi-array average (RMA) was performed by using the justRMA function in *affy *package, which is a part of the BioConductor project [27,28]. For those genes with raw intensities from multiple probes and replicates, we first filtered the outliers by the interquartile range (IQR) rule and retained data between the upper and lower quartiles. Then, the average of all reserved replicates was calculated to represent the expression level for each gene in given conditions.

### Collection of annotation files

EXPath offers gene general information including descriptions, cDNA and protein sequences, Pfam protein families, GO terms, and involved pathways for users' reference. The annotation files of *Arabidopsis thaliana*, *Oryza sativa*, and *Zea mays *were downloaded from TAIR10, RAP-DB, and MaizeGDB separately [29-31]. For descriptions, cDNA and protein sequences, and Pfam protein families, except the descriptions of *Arabidopsis thaliana *were from TAIR10 and the descriptions and sequences of *Oryza sativa *were from RAP-DB, other datasets were acquired by using Ensembl BioMarts [32]. The latest GO terms and involved pathways were collected from gene ontology consortium and KEGG database [33,34].

### Comparative expression analysis

#### Differentially expressed genes

To determine genes that are differentially expressed under given conditions, t-test statistic method was applied by using function t.test() of R package in EXPath. Users can specify a treatment from biotic stress, abiotic stress, or hormone secretion that are well-categorized for three model plants, and then set the time point, fold change and p-value cutoffs. Statistics of fold change and DEG lists (up-regulated and down-regulated) are also provided in EXPath.

#### Coexpression gene groups

Co-expressed genes are a group of genes that express simultaneously under specific conditions. Theoretically, they tend to be controlled under similar transcriptional regulation and involve in identical biological processes or pathways. To investigate this concept, we calculated the coexpression levels of 111 KEGG pathways with number of genes more than 10 by using Pearson's correlation coefficient (PCC) in *Arabidopsis thaliana*. Among them, 92.6% of pathways are positively correlated with satisfied PCCs (most of them are between 0.6 ~ 0.9, see Figure S1, Additional file 1), which suggest that genes involved in the same pathway are generally co-expressed. In EXPath, Pearson's correlation coefficient and Spearman's rank correlation coefficient are applied by using cor() functions in R package to identify genes with co-expression patterns. Normalized raw intensities of genes without log transformation were used to calculate correlation coefficient because it may alter original expression levels that we mentioned previously [35]. Users can customize positive/negative correlation and the conditions (abiotic stress, biotic stress, hormone treatment, and overall conditions) they intend to explore. The expression patterns of coexpression gene groups are illustrated based on z-score transformation:

$$z=\frac{x-\mu }{\sigma }$$

The character z denotes z-score in the above formula, whereas x, μ, and σ represent the raw intensity, mean, and standard deviation of gene expression levels respectively.

#### Enriched KEGG pathways and GO terms

As we know, given a gene list involved in significant biological processes, signal transduction, or metabolic pathways, the pathogenicity of diseases and the roles of these genes can be inferred. It helps researchers to determine biomarkers or treatments for specific diseases. Here, the cumulative probability (p-value) of hypergeometric distribution was calculated to evaluate the KEGG/GO enrichment of a group of input genes. The formula is as follows:

$$p\left(X\leq k\right)=\overset{n}{\underset{i=x}{\sum }}\frac{\left(\begin{array}{c} M\\ i \end{array}\right)\left(\begin{array}{c} N-M\\ n-i \end{array}\right)}{\left(\begin{array}{c} N\\ n \end{array}\right)}$$

where N and M denote the number of background genes and total genes involved in specific KEGG pathways or GO terms, whereas *i *genes out of *n *genes in the gene group × belong to that KEGG pathways or GO terms. The usage of dhyper () and phyper () in R were applied to obtain hypergeometric p-values for each gene group.

## Utility and discussion

### Basic implement in EXPath

**Figure 3:**
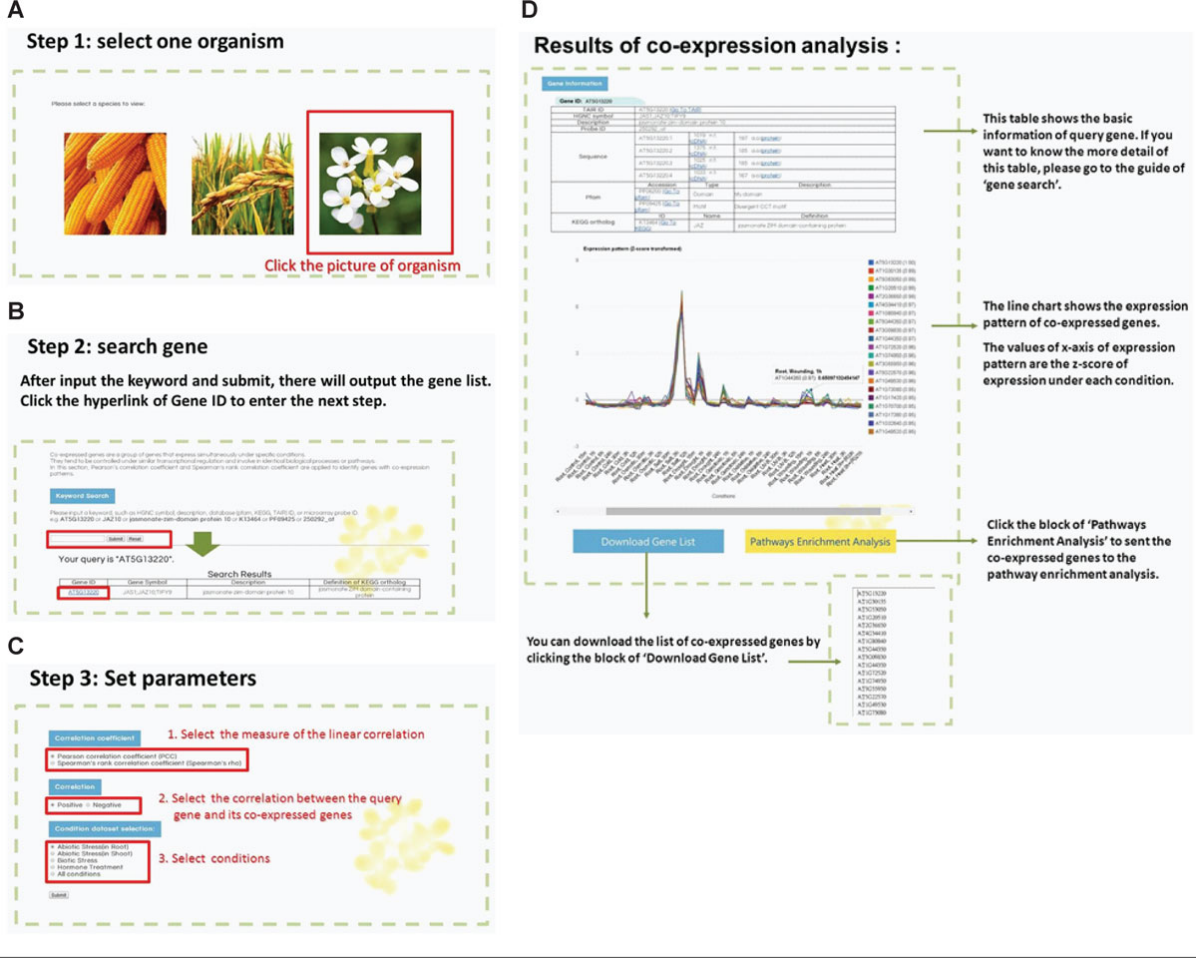
**The output result of "Coexpression analysis" in EXPath**.

EXPath offers a user-friendly interface for exploring Gene Search, Pathway Search, DEGs Search, Pathways/GO Enrichment, and Coexpression analysis. The introduction and guideline for users can be retrieved from the main page. After selecting the EXPath function of interest, users have to specify a model plant first (Figure 3A). For Gene Search and Coexpression analysis, keywords such as HGNC symbol, description, database (pfam, KEGG, TAIR) ID, microarray probe ID, or sequence are valid as query input. Alternatively, users can utilize Gene Browser to access gene lists of three model plants categorized by chromosome. Figure 3B demonstrates a sketch of search result in Coexpression analysis. Moreover, to start a Pathway Search, users can either input a keyword or browse all available pathways classified by metabolism, genetic information processing, environmental information processing, cellular processes, and organismal systems. Prior to the display of KEGG pathway map, users are obligated to select at least one condition from sample list provided in EXPath. By following up the instructions and procedures of each function, the ultimate output is presented and interpreted systematically by users' request. Figure 3 instances an output of Coexpression analysis.

### Advanced combination analysis in EXPath

In addition to explore five functions provided in EXPath separately, all of them are connected with each other using the linkage buttons or hyperlinks in output webpages. For example, in the Gene Search result page, EXPath not only maps the query gene to KEGG pathways to illustrate the involvement of that gene in the corresponding pathway map with its microarray expression levels under specified conditions but also furnishes the linkage button for performing coexpression analysis. Furthermore, advanced combination analysis, the most practical application in EXPath, exposes the powerful pipeline for comparative expression analysis in plants. By combining DEGs Search with Pathways/GO Enrichment, the differentially expressed genes between control and treatment samples are identified first. Then, users can designate up-regulated genes, down-regulated genes, or all DEGs to perform Pathways/GO Enrichment. The enriched KEGG pathways or GO terms of DEGs helps plant scientists to understand, for instance, the resistance to abiotic stresses, pathogenicity of microbes or viruses, and even hormone treatments. Another combination analysis of Coexpression analysis and Pathways/GO Enrichment aims to distinguish coexpressed genes for exhaustively inferring gene functions and their biological roles. A case study given below describes the details of this application.

### Case study: the JAZ10

**Figure 4:**
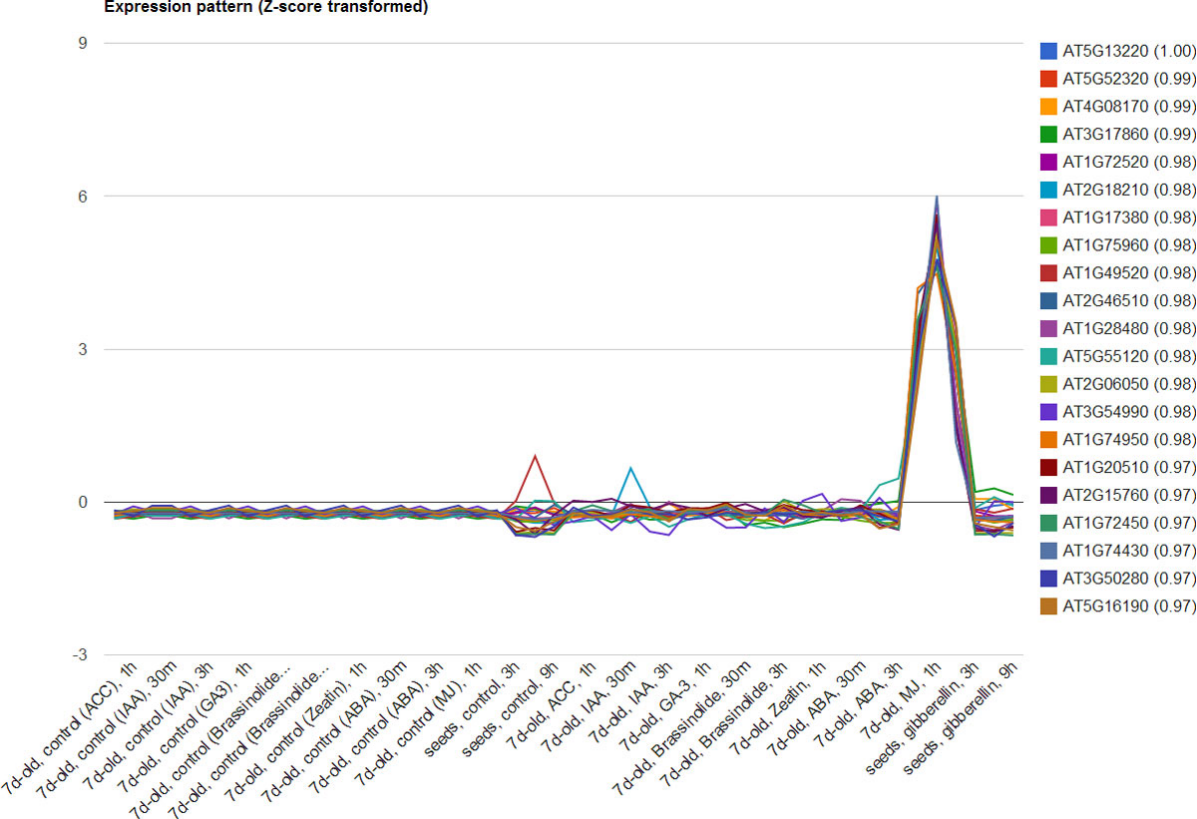
**The expression patterns of JAZ10 and its 165 coexpressed genes**. A dramatic peak appears in the condition of MJ treatment after an hour.

Here, we demonstrate a case study of JASMONATE ZIM-domain protein 10 (JAZ10), which belongs to the TIFY family and is one of the critical repressor in jasmonate signalling [36,37]. Jasmonate (JA) is known to be an essential phytohormone regulating defense mechanisms to pathogens, plant reproductive development, and response to stresses from various environments [38]. Since JAZ family is sensitive to JA treatment, a previous study indicated that the alternative splice variant of JAZ10 plays a significant role in repressing transcription factors that activate the expression of JA response genes in *Arabidopsis *[39]. Based on these valuable findings, we performed the combination approach of Coexpression analysis and Pathways/GO Enrichment to evaluate the usage of EXPath. First of all, 165 coexpressed genes of JAZ10 were identified by setting the parameters of Pearson's correlation coefficient, positive correlation, and hormone treatment (See Table S1, Additional file 2 for coexpressed gene list). Figure 4 shows the z-score transformed expression patterns of top 20 genes coexpressed with JAZ10. A dramatic peak was observed after the methyl jasmonate (MJ) treatment for an hour. Next, the matched genes were submitted to execute pathways and GO enrichment. As expected, both top 10 enriched pathways and GO terms ordered by p-value are consistent with previous findings, e.g., plant-pathogen interaction (Table 2 and 3). It reveals the utility of EXPath for performing comparative expression analysis with high reliability.

**Table 2:** Enriched pathways of JAZ10 coexpressed gene group (partial, only shows top 10 results).

Pathway ID	Pathway name	Hit number (Query)	Percentage in query	P-value
04626	Plant-pathogen interaction	13	29.55%	3.56E-9
00592	alpha-Linolenic acid metabolism	6	13.64%	4.22E-7
00591	Linoleic acid metabolism	3	6.82%	7.45E-5
00400	Phenylalanine, tyrosine and tryptophan biosynthesis	5	11.36%	2.22E-4
00920	Sulfur metabolism	4	9.09%	6.66E-4
04075	Plant hormone signal transduction	8	18.18%	4.16E-3
00966	Glucosinolate biosynthesis	2	4.55%	0.01
00950	Isoquinoline alkaloid biosynthesis	2	4.55%	0.02
01230	Biosynthesis of amino acids	6	13.64%	0.04
00960	Tropane, piperidine and pyridine alkaloid biosynthesis	2	4.55%	0.05

**Table 3:** Enriched GO terms of JAZ10 coexpressed gene group (partial, only shows top 10 results).

GO ID	GO term	Hit number (Query)	Percentage in query	P-value
GO:0009753	response to jasmonic acid	46	29.11%	8.85E-55
GO:0009611	response to wounding	48	30.38%	1.09E-53
GO:0009695	jasmonic acid biosynthetic process	35	22.15%	2.11E-48
GO:0009620	response to fungus	28	17.72%	3.29E-39
GO:0009738	abscisic acid-activated signaling pathway	29	18.35%	7.19E-28
GO:0009867	jasmonic acid mediated signaling pathway	29	18.35%	1.25E-27
GO:0006950	response to stress	85	53.80%	3.71E-27
GO:0007165	signal transduction	59	37.34%	3.62E-26
GO:0009723	response to ethylene	26	16.46%	4.96E-25
GO:0042538	hyperosmotic salinity response	20	12.66%	5.86E-21

## Conclusions

EXPath is an overarching repository geared towards plant scientists to facilitate the retrieval of microarray gene expression data from publicly available resources and the analysis of comparative expression. As the novel database integrating gene expression data with metabolic pathways, the inferred pathways give an insight into the discovery of gene functions, pathogenicity of external invasion, and defense mechanisms for plants. By the usage of five main functions (i.e., Gene Search, Pathway Search, DEGs Search, Pathways/GO Enrichment, and Coexpression analysis) and the advanced combination analysis of them, EXPath indeed provides an effective interface for users to explore the information of interest that will be valuable for further research. Although EXPath facilitates the comparison of expression levels among genes involved in designated pathways, the limited number of plant genes recruited in KEGG database restricts the availability for comparative expression analysis. Another limitation is insufficient expression datasets in public for other plants rather than *Arabidopsis*, rice, and maize. For perspectives, in addition to the expectation of more available plant genes in KEGG database, we will keep surveying any relevant sample with expression profile released in public, especially for those derived from the treatments of biotic stress, abiotic stress, hormone secretion, and even development.

## Availability and requirements

The EXPath database is publicly available at http://EXPath.itps.ncku.edu.tw.

## Competing interests

The authors declare that they have no competing interests.

## Authors' contributions

WCC conceived and designed the experiments and revised the paper. CHC, CNC, and NYW developed the database and webpage. CNC, NYW, YFCH and PFH analyzed the data and performed the experiments. CHC wrote the paper.

## Supplementary Material

Additional file 1 Figure S1. Click here for file

Additional file 2 Table S1. Click here for file

## References

[B1] Atkinson NJ Urwin PE The interaction of plant biotic and abiotic stresses: from genes to the field Journal of experimental botany 2012 63 10 3523 3543 10.1093/jxb/ers100 22467407

[B2] Wang W Vinocur B Altman A Plant responses to drought, salinity and extreme temperatures: towards genetic engineering for stress tolerance Planta 2003 218 1 1 14 10.1007/s00425-003-1105-5 14513379

[B3] Cushman JC Bohnert HJ Genomic approaches to plant stress tolerance Current opinion in plant biology 2000 3 2 117 124 10.1016/S1369-5266(99)00052-7 10712956

[B4] Mittler R Abiotic stress, the field environment and stress combination Trends in plant science 2006 11 1 15 19 10.1016/j.tplants.2005.11.002 16359910

[B5] Rizhsky L Liang H Shuman J Shulaev V Davletova S Mittler R When defense pathways collide. The response of Arabidopsis to a combination of drought and heat stress Plant physiology 2004 134 4 1683 1696 10.1104/pp.103.033431 15047901 PMC419842

[B6] Ren H Gao Z Chen L Wei K Liu J Fan Y Davies WJ Jia W Zhang J Dynamic analysis of ABA accumulation in relation to the rate of ABA catabolism in maize tissues under water deficit Journal of experimental botany 2007 58 2 211 219 16982652 10.1093/jxb/erl117

[B7] Gray WM Hormonal regulation of plant growth and development PLoS biology 2004 2 9 E311 10.1371/journal.pbio.0020311 15367944 PMC516799

[B8] Wang Y Liu C Li K Sun F Hu H Li X Zhao Y Han C Zhang W Duan Y Arabidopsis EIN2 modulates stress response through abscisic acid response pathway Plant molecular biology 2007 64 6 633 644 10.1007/s11103-007-9182-7 17533512

[B9] Boavida LC Borges F Becker JD Feijo JA Whole genome analysis of gene expression reveals coordinated activation of signaling and metabolic pathways during pollen-pistil interactions in Arabidopsis Plant physiology 2011 155 4 2066 2080 10.1104/pp.110.169813 21317340 PMC3091125

[B10] Yonekura-Sakakibara K Tohge T Matsuda F Nakabayashi R Takayama H Niida R Watanabe-Takahashi A Inoue E Saito K Comprehensive flavonol profiling and transcriptome coexpression analysis leading to decoding gene-metabolite correlations in Arabidopsis The Plant cell 2008 20 8 2160 2176 10.1105/tpc.108.058040 18757557 PMC2553606

[B11] Schena M Shalon D Davis RW Brown PO Quantitative monitoring of gene expression patterns with a complementary DNA microarray Science 1995 270 5235 467 470 10.1126/science.270.5235.467 7569999

[B12] Movahedi S Van Bel M Heyndrickx KS Vandepoele K Comparative co-expression analysis in plant biology Plant, cell & environment 2012 35 10 1787 1798 10.1111/j.1365-3040.2012.02517.x 22489681

[B13] Barrett T Wilhite SE Ledoux P Evangelista C Kim IF Tomashevsky M Marshall KA Phillippy KH Sherman PM Holko M NCBI GEO: archive for functional genomics data sets--update Nucleic acids research 2013 41 Database D991 995 23193258 10.1093/nar/gks1193 PMC3531084

[B14] Winter D Vinegar B Nahal H Ammar R Wilson GV Provart NJ An "Electronic Fluorescent Pictograph" browser for exploring and analyzing large-scale biological data sets PloS one 2007 2 8 e718 10.1371/journal.pone.0000718 17684564 PMC1934936

[B15] Dash S Van Hemert J Hong L Wise RP Dickerson JA PLEXdb: gene expression resources for plants and plant pathogens Nucleic acids research 2012 40 Database D1194 1201 22084198 10.1093/nar/gkr938 PMC3245067

[B16] Sato Y Takehisa H Kamatsuki K Minami H Namiki N Ikawa H Ohyanagi H Sugimoto K Antonio BA Nagamura Y RiceXPro version 3.0: expanding the informatics resource for rice transcriptome Nucleic acids research 2013 41 Database D1206 1213 23180765 10.1093/nar/gks1125 PMC3531122

[B17] Zimmermann P Hirsch-Hoffmann M Hennig L Gruissem W GENEVESTIGATOR. Arabidopsis microarray database and analysis toolbox Plant physiology 2004 136 1 2621 2632 10.1104/pp.104.046367 15375207 PMC523327

[B18] Craigon DJ James N Okyere J Higgins J Jotham J May S NASCArrays: a repository for microarray data generated by NASC's transcriptomics service Nucleic acids research 2004 32 Database D575 577 14681484 10.1093/nar/gkh133 PMC308867

[B19] Monaco MK Stein J Naithani S Wei S Dharmawardhana P Kumari S Amarasinghe V Youens-Clark K Thomason J Preece J Gramene 2013: comparative plant genomics resources Nucleic acids research 2014 42 Database D1193 1199 24217918 10.1093/nar/gkt1110 PMC3964986

[B20] Sucaet Y Wang Y Li J Wurtele ES MetNet Online: a novel integrated resource for plant systems biology BMC bioinformatics 2012 13 267 10.1186/1471-2105-13-267 23066841 PMC3483157

[B21] Nikitin A Egorov S Daraselia N Mazo I Pathway studio--the analysis and navigation of molecular networks Bioinformatics 2003 19 16 2155 2157 10.1093/bioinformatics/btg290 14594725

[B22] Tsesmetzis N Couchman M Higgins J Smith A Doonan JH Seifert GJ Schmidt EE Vastrik I Birney E Wu G Arabidopsis reactome: a foundation knowledgebase for plant systems biology The Plant cell 2008 20 6 1426 1436 10.1105/tpc.108.057976 18591350 PMC2483364

[B23] Jensen PA Papin JA Functional integration of a metabolic network model and expression data without arbitrary thresholding Bioinformatics 2011 27 4 541 547 10.1093/bioinformatics/btq702 21172910 PMC6276961

[B24] Beltrame L Bianco L Fontana P Cavalieri D Pathway Processor 2.0: a web resource for pathway-based analysis of high-throughput data Bioinformatics 2013 29 14 1825 1826 10.1093/bioinformatics/btt292 23740747 PMC3702260

[B25] Zheng HQ Chiang-Hsieh YF Chien CH Hsu BK Liu TL Chen CN Chang WC AlgaePath: comprehensive analysis of metabolic pathways using transcript abundance data from next-generation sequencing in green algae BMC genomics 2014 15 196 10.1186/1471-2164-15-196 24628857 PMC4028061

[B26] Kilian J Whitehead D Horak J Wanke D Weinl S Batistic O D'Angelo C Bornberg-Bauer E Kudla J Harter K The AtGenExpress global stress expression data set: protocols, evaluation and model data analysis of UV-B light, drought and cold stress responses The Plant journal: for cell and molecular biology 2007 50 2 347 363 10.1111/j.1365-313X.2007.03052.x 17376166

[B27] Irizarry RA Hobbs B Collin F Beazer-Barclay YD Antonellis KJ Scherf U Speed TP Exploration, normalization, and summaries of high density oligonucleotide array probe level data Biostatistics 2003 4 2 249 264 10.1093/biostatistics/4.2.249 12925520

[B28] Gautier L Cope L Bolstad BM Irizarry RA affy--analysis of Affymetrix GeneChip data at the probe level Bioinformatics 2004 20 3 307 315 10.1093/bioinformatics/btg405 14960456

[B29] Lamesch P Berardini TZ Li D Swarbreck D Wilks C Sasidharan R Muller R Dreher K Alexander DL Garcia-Hernandez M The Arabidopsis Information Resource (TAIR): improved gene annotation and new tools Nucleic acids research 2012 40 Database D1202 1210 22140109 10.1093/nar/gkr1090 PMC3245047

[B30] Sakai H Lee SS Tanaka T Numa H Kim J Kawahara Y Wakimoto H Yang CC Iwamoto M Abe T Rice Annotation Project Database (RAP-DB): an integrative and interactive database for rice genomics Plant & cell physiology 2013 54 2 e6 10.1093/pcp/pcs183 23299411 PMC3583025

[B31] Schaeffer ML Harper LC Gardiner JM Andorf CM Campbell DA Cannon EK Sen TZ Lawrence CJ MaizeGDB: curation and outreach go hand-in-hand Database: the journal of biological databases and curation 2011 2011 bar022 21624896 10.1093/database/bar022 PMC3104940

[B32] Kinsella RJ Kahari A Haider S Zamora J Proctor G Spudich G Almeida-King J Staines D Derwent P Kerhornou A Ensembl BioMarts: a hub for data retrieval across taxonomic space Database: the journal of biological databases and curation 2011 2011 bar030 21785142 10.1093/database/bar030 PMC3170168

[B33] Ashburner M Ball CA Blake JA Botstein D Butler H Cherry JM Davis AP Dolinski K Dwight SS Eppig JT Gene ontology: tool for the unification of biology. The Gene Ontology Consortium Nature genetics 2000 25 1 25 29 10.1038/75556 10802651 PMC3037419

[B34] Kanehisa M Goto S Sato Y Kawashima M Furumichi M Tanabe M Data, information, knowledge and principle: back to metabolism in KEGG Nucleic acids research 2014 42 Database D199 205 24214961 10.1093/nar/gkt1076 PMC3965122

[B35] Chien CH Chiang-Hsieh YF Tsou AP Weng SL Chang WC Huang HD Large-Scale Investigation of Human TF-miRNA Relations Based on Coexpression Profiles BioMed research international 2014 2014 623078 24995316 10.1155/2014/623078 PMC4068100

[B36] Vanholme B Grunewald W Bateman A Kohchi T Gheysen G The tify family previously known as ZIM Trends in plant science 2007 12 6 239 244 10.1016/j.tplants.2007.04.004 17499004

[B37] Chini A Fonseca S Fernandez G Adie B Chico JM Lorenzo O Garcia-Casado G Lopez-Vidriero I Lozano FM Ponce MR The JAZ family of repressors is the missing link in jasmonate signalling Nature 2007 448 7154 666 671 10.1038/nature06006 17637675

[B38] Moreno JE Shyu C Campos ML Patel LC Chung HS Yao J He SY Howe GA Negative feedback control of jasmonate signaling by an alternative splice variant of JAZ10 Plant physiology 2013 162 2 1006 1017 10.1104/pp.113.218164 23632853 PMC3668036

[B39] Chung HS Howe GA A critical role for the TIFY motif in repression of jasmonate signaling by a stabilized splice variant of the JASMONATE ZIM-domain protein JAZ10 in Arabidopsis The Plant cell 2009 21 1 131 145 10.1105/tpc.108.064097 19151223 PMC2648087

