# Supplementary figures and images for: EXPath: a database of comparative expression analysis inferring metabolic pathways for plants
Source: BMC Genomics. 2015 Jan 21;16(Suppl 2):S6. doi: 10.1186/1471-2164-16-S2-S6 (PMC4331720; doi:10.1186/1471-2164-16-S2-S6)

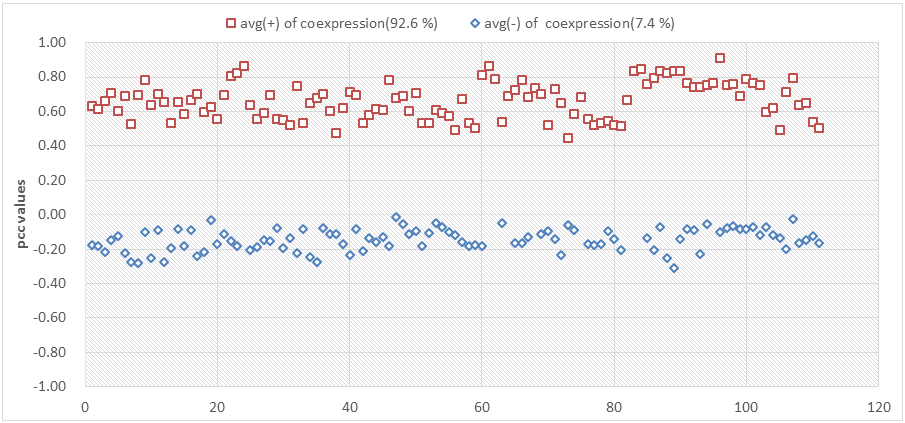

Supplement: Additional file 1 — Figure S1. [file 1471-2164-16-S2-S6-S1.bmp]
