# Supplementary material for: EXPath: a database of comparative expression analysis inferring metabolic pathways for plants
Source: BMC Genomics. 2015 Jan 21;16(Suppl 2):S6. doi: 10.1186/1471-2164-16-S2-S6 (PMC4331720; doi:10.1186/1471-2164-16-S2-S6)
Supplement: Additional file 2 — Table S1. [file 1471-2164-16-S2-S6-S2.pdf]

Table S1 165 coexpressed genes of JAZ10 identified by Coexpression analysis in EXPath.

TAIR ID	Description
AT1G06620	2-oxoglutarate (2OG) and Fe(II)-dependent oxygenase superfamily protein
AT1G09090	respiratory burst oxidase homolog B
AT1G12240	Glycosyl hydrolases family 32 protein
AT1G14120	2-oxoglutarate (2OG) and Fe(II)-dependent oxygenase superfamily protein
AT1G14130	2-oxoglutarate (2OG) and Fe(II)-dependent oxygenase superfamily protein
AT1G16370	organic cation/carnitine transporter 6
AT1G16670	Protein kinase superfamily protein
AT1G17380	jasmonate-zim-domain protein 5
AT1G17420	lipoxygenase 3
AT1G17750	PEP1 receptor 2
AT1G18710	myb domain protein 47
AT1G19180	jasmonate-zim-domain protein 1
AT1G19300	Nucleotide-diphospho-sugar transferases superfamily protein
AT1G19670	chlorophyllase 1
AT1G20490	AMP-dependent synthetase and ligase family protein
AT1G20510	OPC-8:0 CoA ligase1
AT1G20823	RING/U-box superfamily protein
AT1G23850	unknown protein
AT1G24100	UDP-glucosyl transferase 74B1
AT1G25500	Plasma-membrane choline transporter family protein
AT1G26730	EXS (ERD1/XPR1/SYG1) family protein
AT1G27730	salt tolerance zinc finger
AT1G28050	B-box type zinc finger protein with CCT domain
AT1G28480	Thioredoxin superfamily protein
AT1G30135	jasmonate-zim-domain protein 8
AT1G32640	Basic helix-loop-helix (bHLH) DNA-binding family protein
AT1G32970	Subtilisin-like serine endopeptidase family protein
AT1G42540	glutamate receptor 3.3
AT1G44350	IAA-leucine resistant (ILR)-like gene 6
AT1G49520	SWIB complex BAF60b domain-containing protein
AT1G50460	hexokinase-like 1
AT1G58200	MSCS-like 3
AT1G60260	beta glucosidase 5
AT1G60270	beta glucosidase 6

AT1G64200	vacuolar H+-ATPase subunit E isoform 3
AT1G65860	flavin-monooxygenase glucosinolate S-oxygenase 1
AT1G69370	chorismate mutase 3
AT1G70700	TIFY domain/Divergent CCT motif family protein
AT1G72450	jasmonate-zim-domain protein 6
AT1G72470	exocyst subunit exo70 family protein D1
AT1G72520	PLAT/LH2 domain-containing lipoxygenase family protein
AT1G73080	PEP1 receptor 1
AT1G74100	sulfotransferase 16
AT1G74420	fucosyltransferase 3
AT1G74430	myb domain protein 95
AT1G74950	TIFY domain/Divergent CCT motif family protein
AT1G75300	NmrA-like negative transcriptional regulator family protein
AT1G75960	AMP-dependent synthetase and ligase family protein
AT1G76070	unknown protein
AT1G76380	DNA-binding bromodomain-containing protein
AT1G76600	unknown protein
AT1G76640	Calcium-binding EF-hand family protein
AT1G76650	calmodulin-like 38
AT1G79310	metacaspase 7
AT1G80840	WRKY DNA-binding protein 40
AT2G06050	oxophytodienoate-reductase 3
AT2G15760	Protein of unknown function (DUF1645)
AT2G17330	Expressed pseudogene
AT2G18210	unknown protein
AT2G19780	Leucine-rich repeat (LRR) family protein
AT2G22010	related to KPC1
AT2G22200	Integrase-type DNA-binding superfamily protein
AT2G22760	basic helix-loop-helix (bHLH) DNA-binding superfamily protein
AT2G22770	basic helix-loop-helix (bHLH) DNA-binding superfamily protein
AT2G22850	basic leucine-zipper 6
AT2G22860	phytosulfokine 2 precursor
AT2G24850	tyrosine aminotransferase 3
AT2G25735	unknown protein
AT2G26530	Protein of unknown function (DUF1645)
AT2G27310	F-box family protein
AT2G27690	cytochrome P450, family 94, subfamily C, polypeptide 1
AT2G29440	glutathione S-transferase tau 6

AT2G29450	glutathione S-transferase tau 5
AT2G31180	myb domain protein 14
AT2G31230	ethylene-responsive element binding factor 15
AT2G32150	Haloacid dehalogenase-like hydrolase (HAD) superfamily protein
AT2G32400	glutamate receptor 5
AT2G34070	TRICHOME BIREFRINGENCE-LIKE 37
AT2G34600	jasmonate-zim-domain protein 7
AT2G38240	2-oxoglutarate (2OG) and Fe(II)-dependent oxygenase superfamily protein
AT2G38760	annexin 3
AT2G42760	unknown protein
AT2G44840	ethylene-responsive element binding factor 13
AT2G44940	Integrase-type DNA-binding superfamily protein
AT2G46510	ABA-inducible BHLH-type transcription factor
AT2G47950	unknown protein
AT3G06500	Plant neutral invertase family protein
AT3G08990	Yippee family putative zinc-binding protein
AT3G09830	Protein kinase superfamily protein
AT3G09940	monodehydroascorbate reductase
AT3G09960	Calcineurin-like metallo-phosphoesterase superfamily protein
AT3G10260	Reticulon family protein
AT3G10930	unknown protein
AT3G14050	RELA/SPOT homolog 2
AT3G15500	NAC domain containing protein 3
AT3G16350	Homeodomain-like superfamily protein
AT3G17120	unknown protein
AT3G17860	jasmonate-zim-domain protein 3
AT3G22160	VQ motif-containing protein
AT3G22400	PLAT/LH2 domain-containing lipoxygenase family protein
AT3G25780	allene oxide cyclase 3
AT3G44260	Polynucleotidyl transferase, ribonuclease H-like superfamily protein
AT3G44720	arogenate dehydratase 4
AT3G50130	Plant protein of unknown function (DUF247)
AT3G50280	HXXXD-type acyl-transferase family protein
AT3G51450	Calcium-dependent phosphotriesterase superfamily protein
AT3G53600	C2H2-type zinc finger family protein
AT3G54990	Integrase-type DNA-binding superfamily protein
AT3G55950	CRINKLY4 related 3
AT3G56200	Transmembrane amino acid transporter family protein

AT3G59130	Cysteine/Histidine-rich C1 domain family protein
AT3G59710	NAD(P)-binding Rossmann-fold superfamily protein
AT3G61400	2-oxoglutarate (2OG) and Fe(II)-dependent oxygenase superfamily protein
AT3G62010	unknown protein
AT4G08170	Inositol 1,3,4-trisphosphate 5/6-kinase family protein
AT4G10390	Protein kinase superfamily protein
AT4G14680	Pseudouridine synthase/archaeosine transglycosylase-like family protein
AT4G14940	amine oxidase 1
AT4G15330	cytochrome P450, family 705, subfamily A, polypeptide 1
AT4G15440	hydroperoxide lyase 1
AT4G21680	NITRATE TRANSPORTER 1.8
AT4G22610	Bifunctional inhibitor/lipid-transfer protein/seed storage 2S albumin superfamily protein
AT4G24350	Phosphorylase superfamily protein
AT4G29700	Alkaline-phosphatase-like family protein
AT4G36500	unknown protein
AT4G37710	VQ motif-containing protein
AT4G37850	basic helix-loop-helix (bHLH) DNA-binding superfamily protein
AT4G39030	MATE efflux family protein
AT4G39940	APS-kinase 2
AT4G39980	3-deoxy-D-arabino-heptulosonate 7-phosphate synthase 1
AT5G02940	Protein of unknown function (DUF1012)
AT5G05600	2-oxoglutarate (2OG) and Fe(II)-dependent oxygenase superfamily protein
AT5G05730	anthranilate synthase alpha subunit 1
AT5G06870	polygalacturonase inhibiting protein 2
AT5G12340	unknown protein
AT5G13220	jasmonate-zim-domain protein 10
AT5G14700	NAD(P)-binding Rossmann-fold superfamily protein
AT5G16190	cellulose synthase like A11
AT5G19100	Eukaryotic aspartyl protease family protein
AT5G19110	Eukaryotic aspartyl protease family protein
AT5G19450	calcium-dependent protein kinase 19
AT5G22250	Polynucleotidyl transferase, ribonuclease H-like superfamily protein
AT5G23580	calmodulin-like domain protein kinase 9
AT5G23810	amino acid permease 7
AT5G24290	Vacuolar iron transporter (VIT) family protein
AT5G40210	nodulin MtN21 /EamA-like transporter family protein
AT5G44350	ethylene-responsive nuclear protein -related
AT5G47240	nudix hydrolase homolog 8

AT5G47910	respiratory burst oxidase homologue D
AT5G52120	phloem protein 2-A14
AT5G52320	cytochrome P450, family 96, subfamily A, polypeptide 4
AT5G52430	hydroxyproline-rich glycoprotein family protein
AT5G53050	alpha/beta-Hydrolases superfamily protein
AT5G55120	galactose-1-phosphate guanylyltransferase (GDP)s;GDP-D-glucose phosphorylases;quercetin 4'-O-glucosyltransferases
AT5G56760	serine acetyltransferase 1;1
AT5G56980	unknown protein
AT5G59730	exocyst subunit exo70 family protein H7
AT5G60890	myb domain protein 34
AT5G63450	cytochrome P450, family 94, subfamily B, polypeptide 1
AT5G63970	Copine (Calcium-dependent phospholipid-binding protein) family
AT5G63980	Inositol monophosphatase family protein
AT5G64900	precursor of peptide 1
AT5G66650	Protein of unknown function (DUF607)
AT5G67150	HXXXD-type acyl-transferase family protein
AT5G67210	Protein of unknown function (DUF579)
